# Influence of Work-Related and Personal Characteristics on the Burnout Risk among Full- and Part-Time Teachers

**DOI:** 10.3390/ijerph18041535

**Published:** 2021-02-05

**Authors:** Reingard Seibt, Steffi Kreuzfeld

**Affiliations:** Institute for Preventive Medicine, Rostock University Medical Centre, 18055 Rostock, Germany; reingard.seibt@uni-rostock.de

**Keywords:** teachers, burnout risk, effort-reward imbalance, overcommitment, inability to recover

## Abstract

Teachers are at increased risk of stress-related illnesses and burnout symptoms. Thus, a cross-sectional study involving 6109 full-time and 5905 part-time teachers at upper-level secondary schools examined the influence of presumed work-related and personal characteristics on burnout risk between January and April 2018. Burnout was recorded using the Maslach Burnout Inventory—General Survey (MBI-GS). Work-related characteristics were weekly working hours and work stress, operationalized with the effort-reward imbalance (ERI) model. Overcommitment and the inability to recover were determined as personal characteristics. Multiple linear regression analyses were performed, adjusted for age and gender. Overall, 47% of the teachers reported burnout symptoms and 3% had an indication of burnout. Full-time and part-time teachers did not differ in their risk of burnout. ERI, overcommitment, and inability to recover were identified as predictors of burnout risk (explained variance: 29%), whereby the inability to recover was the strongest predictor. In contrast, weekly working hours, extent of employment, gender and age were not related to the burnout risk. ERI was found in 33%, inability to recover in 36% and overcommitment in 39% of all the teachers studied. In particular, the inability to recover should be taken into account as an early indicator of burnout.

## 1. Introduction

The teaching profession is characterised by a diverse range of tasks under complex working conditions, in which high work demands and psychosocial interactions with students, parents and colleagues dominate as potential stressors [1,2,3,4]. Compared to other professional groups, teachers are more affected by work stress, anxiety, fatigue and sleep problems [5,6,7]. In addition, there is an increased risk of stress-related mental and psychosomatic illnesses and burnout [8,9].

In the International Classification of Diseases (ICD-11), burnout is not viewed as a disease but in the sense of a work-related phenomenon as the result of an unsuccessful handling of chronic, work-related stress [10]. The process-like course of burnout is typical: after initial phases of overload, psychosomatic disorders can develop into physical and psychological exhaustion and depression [3].

One of the most frequently cited definitions of burnout syndrome goes back to Maslach and Jackson [11]. According to this definition, burnout syndrome comprises the following three dimensions: emotional exhaustion, depersonalisation/cynicism and personal accomplishment. High values in the emotional exhaustion and depersonalisation/cynicism dimensions as well as low values in the personal accomplishment dimension are characteristic of burnout [12]. While high emotional exhaustion and low personal performance are typical reactions to stress, depersonalisation/cynicism is seen more as a coping strategy (creating distance).

The prevalence rates for burnout among teachers fluctuate between 0 and 71% [8,13], which can also be attributed to the different concepts and the selected survey method. Representative data on the incidence of burnout syndrome for German teachers are not yet available [14].

Previous burnout research has assumed that both structural causes in working conditions as well as individual factors play a role in the development of the syndrome [15,16,17,18,19]. The most important work organisational causes of burnout include, on the one hand, high job demands such as high workload, work pressure, and role conflicts and, on the other hand, a lack of professional resources such as a lack of support from colleagues and superiors, and workplace injustice [15,18,20]. Both high work demand and a lack of professional resources can have a negative impact on mental health and well-being and increase the occurrence of burnout symptoms (see for review [21]). The study by Hultell and Gustavsson [22] provided evidence that work requirements are more strongly related to burnout risk than professional resources, which are more strongly related to work engagement.

The effort-reward imbalance (ERI) model [23] among other models has been successfully used to assess the effects of work-related stress on health [24,25,26]. Compared to other methods, the ERI model takes into account both extrinsic and intrinsic factors when assessing the effects of psychosocial outcomes of health [27]. An imbalance of high professional effort (requirements, obligations) and low reward (money, esteem, career opportunities, and job security) is considered to be a non-specific factor for an increased, stress-associated risk of illness, especially for the development of depression and burnout [26,27,28].

The intrinsic component in the ERI model is the coping pattern “overcommitment” (OC). It describes an individual coping style with the tendency to exhaust oneself regardless of one’s own resources. OC is a predictor of anxiety and depression [27,29].

The extent to which the above-mentioned structural and work organisational factors affect the individual dimensions of burnout has been the subject of numerous studies, illustrating that occupational control in particular as well as social support and justice in the workplace have a protective effect on the level of emotional exhaustion [30]. In contrast, high demands, high workload, low reward and job insecurity increase the risk of emotional exhaustion. The cynicism dimension has been associated with most of the work-related factors examined. In contrast, the personal performance dimension has only been associated with low reward [30].

High workload can also result from long working hours, for instance weekly work volumes of more than 40 h [31]. They are also associated with an increased risk of depressive states, anxiety, and adverse sleeping conditions [32]. The risk of work stress and burnout increases with the number of hours per week [33,34]. In Germany, in 2019, around 40% of all upper-secondary level schoolteachers worked part-time. 80% of them were women [35].

The effect of the extent of employment on mental health or the risk of burnout seems to be rather unexplained. This also applies to random samples of teachers, for which only a few comparative studies between full-time and part-time teachers have been available so far [25,36]. The study by Unterbrink et al. [25] determined a lower personal efficacy for part-time upper-level secondary schoolteachers. Seibt et al. [36] found no differences between full-time and part-time teachers regarding mental health, nor was there a relationship between working conditions and health status. Other studies have shown that part-time teachers feel more stressed than full-time teachers and are less able to recover sufficiently [37,38].

One explanation for this could be that the time for adequate recovery decreases with the number of weekly working hours. Recovery after work (external recovery) is particularly important if there are insufficient opportunities during work (internal recovery) [39]. This is regularly the case for teachers. Long working hours hinder subsequent recovery processes through physiological activation which persists during working hours [40,41,42]. Stress-related cognitive processes such as rumination influence necessary recovery processes [43,44,45]. Psychosomatic reactions like pain also contribute to the fact that recovery in leisure time is restricted [46]. In summary, it can be stated that sustained physiological activation and incomplete recovery are important precursors for work stress and burnout symptoms.

In addition to being able to recover sufficiently, people must also have the ability to recover. Thus, the inability to recover is viewed as an individual coping pattern in dealing with the demands of work [47]. In studies, the inability to recover has repeatedly been found to be a risk factor for teachers’ mental health [37,38].

The aim of this study was to investigate the influence of work-related (working hours, effort-reward ratio) and personal characteristics (inability to recover, overcommitment) on the risk of burnout among full-time and part-time teachers. The second aim was to clarify which of these characteristics are predictors of burnout risk and whether part-time teachers have a lower risk of burnout compared to full-time teachers.

The results are expected to provide insights into avoidable risk factors as well as resources that need to be implemented early on to maintain mental health.

## 2. Materials and Methods

### 2.1. Procedure

The study on the estimation of working time, workload and health of upper-level secondary school teaching staff (LaiW study) was conducted as a full survey between January and April 2018 in all sixteen federal states of Germany. A study period with an average workload was selected for each federal state in order to ensure comparable working conditions nationwide.

Voluntary participation in the study was advertised in advance through posters and flyers at all upper-level secondary schools in Germany. Immediately before the start of the study, all teachers at these schools received an information letter which explained the conditions for participation and access to the study, data protection, and the conduct of the study and data analysis. The anonymity of the data was guaranteed by transaction numbers (TANs) and an eight-digit personal code that was only known to the participants themselves. The data were recorded via an online portal at the University of Rostock. 

A list of answers to frequently asked questions (FAQ) was then made available on the study website. In addition, there was the possibility of telephone and electronic queries to the team of investigators over the entire investigation period.

First, all participants answered the questions in the online questionnaire (OQ) once. Then, they documented their daily working hours over a period of four weeks (28 days) using defined activity categories in an online protocol (OP). 

### 2.2. Participants

A total of more than 20,000 upper-level secondary school teachers (hereafter: teachers) took part in the LaiW study. Of those, 18,791 filled in the OQ completely and 14,338 participants filled in the OQ and the OP completely. Since the proportion of teaching hours, the main tasks of teachers, is relevant to assess their working time, only teachers with up to a three-hour reduction in teaching hours were included in the sample. Thus, the sample consisted of 6109 full-time (FT-T) and 5905 part-time (PT-T) teachers. Any employment that is less than 100% of full-time employment is considered part-time. The characteristics of the sample are presented in Table 1. 

The sample consisted of approximately one third men (32%) and two-thirds women (68%). As expected, the number of women was higher for part-time teachers than for full-time teachers (81% vs. 56%), corresponding to the gender division of teachers in Germany.

Full-time teachers are three years younger (average of 41 ± 10 years old) than the part-time teachers (44 ± 9 years old, *p* < 0.001). Half of the full-time and about one third of the part-time teachers was younger than 40 years old. Approximately one quarter of the participants was older than 50 years. 

The most common subjects taught were languages, combinations of languages and social sciences, and natural sciences, whereby subjects with only languages occur more often for part-time teachers.

Approximately 6% of teachers indicated that they cared for relatives at home. Children at home were looked after by approx. three quarters (73%) of part-time teachers, but only one third of full-time teachers (35%). Most teachers indicated they lived in a steady relationship or with a permanent partner (85%).

### 2.3. Instruments

#### 2.3.1. Online-Protocol (OP) for Recording Working Time

The average weekly working time was determined using the OP over 28 days. The teachers documented their working hours on a daily basis using 12 teacher-specific activity categories: number of lessons and substitute lessons, preparation and follow-up time of lessons, correction and grading of pupils’ work, implementation of projects and excursions, extracurricular work with pupils (including integration and inclusion), co-operation with parents, teamwork with colleagues, administrative tasks, supervision, and all other tasks.

To determine the total weekly working time (WWT), the average WWT was calculated for each activity category. The amount of time for the individual activity categories was previously examined for statistical outliers. Extreme values within the individual activity categories were replaced by subject-specific mean values.

#### 2.3.2. Online-Questionnaire (OQ) for Recording Work-Related and Personal Characteristics

In addition to socio-demographic (gender, age, family status, etc.) and professional data (teaching requirement, reduction in hours for special tasks, subjects taught, classes, number of pupils, etc.), the OQ contained questions on work-related and personal characteristics as well as burnout risk [48,49]. Work-related characteristics were the volume of employment as well as the subscales of the Effort-Reward Imbalance Questionnaire (ERI-Q) [23]. Personal characteristics included were inability to recover [47] and overcommitment (ERI-Q) [23].

#### 2.3.3. Maslach Burnout Inventory—General Survey (MBI-GS)

The most widely used instrument for measuring burnout is the Maslach Burnout Inventory (MBI) [50]. It measures the incidence of burnout symptoms from never to daily. To calculate the burnout risk the German translation of the MBI-GS [49] with the three burnout subscales emotional exhaustion (5 items), depersonalisation/cynicism (5 items) and personal accomplishment (6 items) were used. Each of these 16 items was assessed on a seven-point Likert scale (0 = never up to 6 = daily) corresponding to the frequency of occurrence. The mean values were formed for each of the three burnout subscales. A burnout syndrome is assumed when emotional exhaustion and cynicism are high, but personal accomplishment is low [49,50].

However, since no reliable statement about the burnout syndrome could be made from the mean values of the three subscales, the scale values according to Kalimo et al. [48] were weighted and summarised to the following burnout score: burnout score = (0.4 × exhaustion) + (0.3 × cynicism) + (0.3 × performance). The burnout risk could then be determined on the basis of this burnout score: if the value was below 1.49, there was no indication of burnout, in the range from 1.50 to 3.49 points there were some burnout symptoms and from the value 3.50 upwards there were indications of burnout syndrome [48].

The validity of the MBI has been demonstrated for normal and clinical populations [12] as well as for different occupational groups and cultures [51]. In a sample of 1316 persons, Maslach and Jackson [52] indicated internal consistencies in the form of Cronbach’s alphas for emotional exhaustion of 0.90, for depersonalisation/cynicism of 0.79 and for personal accomplishment of 0.71. For the MBI-GS, Schaufeli et al. [49] reported Cronbach’s alphas from 0.87 (emotional exhaustion) to 0.64 (depersonalisation) and 0.70 (personal accomplishment) and thus good to satisfactory Cronbach’s alpha values. For the three burnout subscales of the study presented here, Cronbach’s alpha is between 0.79 and 0.84 and, thus, according to Blanz [53], in the range of acceptable or good.

#### 2.3.4. Effort-Reward-Imbalance Questionnaire (ERI-Q)

The short version of the ERI-Q developed by Siegrist et al. [23] comprised the main scales effort (three items; range: 3–15 points), reward (seven items; range: 7–35 points), and the effort-reward ratio (ER ratio). The reward scale was made up of the three subscales status or professional advancement, appreciation or recognition, and job security. High total values indicated high levels of perceived effort or reward.

The ER ratio was formed from the sum values of the two main subscales using the following rule: ER ratio = ∑ effort/(∑ reward × 0.54). An ER ratio of >1 indicated an effort-reward imbalance (ERI) [23,54], which is associated with a health risk. The greater the imbalance between effort and reward, the greater the health risk.

The validity and reliability of the German ERI questionnaire were satisfactory. For all subscales of the short version of the ERI-Q [23], the values of the internal consistency were above α = 0.70 (effort: 0.74, reward: 0.79). For the ER scales of this study, lower Cronbach alphas of 0.61 (effort) and 0.72 (reward) were determined, which can be assigned to the questionable or acceptable range 53. For the subscale effort (three items), it should be noted that the Cronbach’s alpha inevitably decreases with a falling number of items [55].

#### 2.3.5. Overcommitment (OC)

The intrinsic component of the ERI-Q overcommitment (six items) was recorded on a four-point Likert scale (1 = strongly disagree to 4 = strongly agree). A total score (OC score) was also formed from the six items on this scale (range: 6–24 points). High values on the OC scale meant that there was a high intrinsic tendency to exert oneself. The upper tercile of the total score was defined as the risk group [56].

For the subscale of overcommitment, Cronbach’s Alpha was given as 0.79 [23,56]. In the present LaiW study, an acceptable Cronbach’s alpha of 0.77 was determined for overcommitment [53].

#### 2.3.6. Inability to Recover (IR)

The questionnaire for the analysis of stress-relevant coping requirements (German: FABA) [47] recorded the inability to relax, indicating that an extreme work commitment is associated with an accepted limited ability to recover in the sense of an inefficient coping style [57].

The inability to recover as a subscale of the FABA [47] was recorded with six items on the basis of a four-point ranking scale (1 = does not apply at all to 4 = strongly applies). Then, the total score (IR score—range: 6–24 points) was calculated from the six items, which could be assigned to normal (6–18 points), unable to recover (19–21 points) and very unable to recover (22–24 points) using percentile values.

The reliability of the IR factor was rated as good; Cronbach’s α was 0.79 [58]. In the present study, a Cronbach alpha also of 0.79 was determined for IR, which was considered acceptable and was at the limit of the good range [53].

### 2.4. Data Analyses

#### 2.4.1. Quality Management of Data Processing

In both the OQ and in the OP, input aids and default settings prevented implausible time entries. Only study participants for whom both an OQ and an OP were available were included in the data analysis. Using the personal code, both documents could be merged for data evaluation. The completeness of the information in the OP was then checked. Participants who recorded their working hours on fewer than 21 out of 28 days were not included in the data analysis. 

Prior to the statistical calculations, the entire dataset was checked for implausible information. The number of teaching hours and the reduction in hours (reduced teaching time) was verified in the OQ on the basis of information on age and the special tasks of the teacher. The range of time for the individual activity categories was examined in the OP for statistical outliers every 28 days. Extreme values were replaced by subject-specific mean values within the individual activity categories.

The times in the activity categories in the OQ and in the OP were indicated in minutes—with the exception of the teaching and substitute lessons (45-min units). To determine the total weekly working time, all information was converted into hours. In the OP, the average values over 4 weeks were calculated for all activity categories, provided there were no sick days (74% of the full-time data records). If there were any sick days documented, these weeks were not taken into account when calculating the average weekly working time. Instead, the average value was calculated from the remaining weeks without sick days. During the investigation period, 10% of the participating full-time teachers were sick for one day, another 14% were sick for two to five days and 2% were absent from work for six to ten days due to illness.

For the work-related and personal characteristics (MBI-GS, ERI-Q, OC, IR), the values belonging to the selected responses were automatically transferred to the database. Hardly any corrections were necessary here in the case of only small errors. Missing values were supplemented by singular imputation.

#### 2.4.2. Statistics

The statistical analysis of the data was carried out with the programme Statistical Package for Social Science version 27.0 (IBM SPSS, Inc., Chicago, IL, USA). Differences between full-time and part-time teachers were examined—adjusted for gender and age—using univariate and multivariate covariance analyses (MANOVA).

The correlation coefficient according to Pearson (r) and the rank correlation coefficient according to Spearman (R) were used to test the relationship between work-related and personal characteristics, and the burnout risk. The influence of gender and age on these associations was also adjusted using partial correlations. Correlation coefficients r < ±0.10 were interpreted as being independent of one another.

In all statistical tests, a result with a probability of error of *p* < 0.05 was considered significant. In order to estimate the practical relevance, effect sizes (partial eta-square η^2^_partial_) were also determined in the variance analyses and interpreted according to the conventions of Cohen [59]. This led to statistically significant effects of a small effect size of η^2^ = 0.01 (d = 0.20) being considered relevant in the variance analyses.

Linear regression analyses were carried out to examine the influence on the burnout risk (criterion variables) of the work-related and personal characteristics as well as the control variables (independent variables), in order to clarify which of the characteristics examined were relevant predictors of the burnout risk in teachers. To do this, each work-related and personal characteristic was subjected to a linear single regression in the first step of the analysis and to a multiple linear regression in the second step. Finally, an overall model (method: inclusion) was created which contained all the significant characteristics. In addition, the coefficient of determination R^2^ was determined for the quality of the model (goodness-of-fit).

## 3. Results

### 3.1. Burnout Risk of Full-Time and Part-Time Teachers

Significant differences were found between full-time and part-time teachers (*p* = 0.32–<0.001) for the burnout scales [49]—adjusted for gender and age. However, they were practically irrelevant (η^2^ < 0.010) (Table 2). 

The average values of the teachers (range: 0–6 points) are for emotional exhaustion 2.3 points, for cynicism 1.2 points and for professional accomplishment 4.8 points (Table 2). This means that on average the teachers in this study experienced emotional exhaustion “once a month”, and cynicism and personal accomplishment only “once a year” on average. These results are not influenced by gender and age effects (η^2^ = 0.003–<0.001) (Table 2), which means that neither the burnout subscales (C = 0.08–0.09) nor the burnout risk (C = 0.04) are related to gender. Age does not correlate with the burnout subscales (r = 0.03–0.17) and nor does burnout risk (r = 0.10), or only very slightly. 

**Figure 1:**
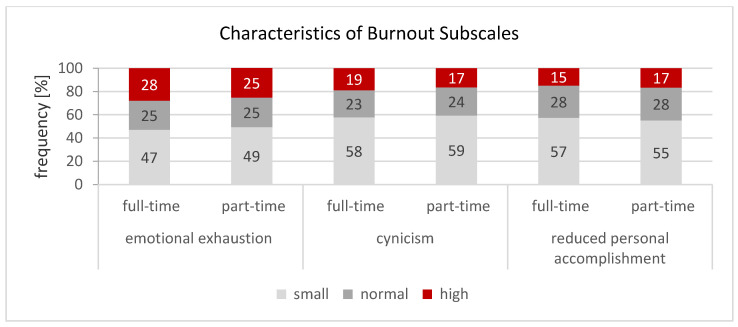
Characteristics of burnout subscales of full-time (*n* = 6109) and part-time teachers (*n* = 5905). Comments: χ^2^-test (Pearson); χ^2^: test size; significance (two-sided); d: effect size (emotional exhaustion: *p* = 0.007, d = 0.057, no effect; cynicism: *p* = 0.002, d = 0.064, no effect; reduced personal accomplishment: *p* < 0.001, d = 0.104, no effect) [59].

The expression of the burnout subscales also did not differ in a practically significant way between full-time and part-time teachers (Figure 1). Around a quarter of all teachers recorded high levels of exhaustion, 18% reported high levels of cynicism and 17% reported poor professional efficacy.

The burnout risk was determined according to the formula and evaluation criteria of Kalimo et al. [48] (see Section 2.3.3). Based on this, again no differences were found between full-time and part-time teachers for burnout risk (*p* = 0.055) (Table 3). While no risk of burnout could be determined in this study for almost half of the teachers (FT-T: 49%, PT-T: 50%), 47% of all teachers showed *some burnout symptoms* and 3% of them indications of *burnout*. 

A burnout syndrome is assumed when emotional exhaustion and cynicism are high, but personal accomplishment is low. Burnout is present when all three burnout criteria are fulfilled.

### 3.2. Work-Related Characteristics for Full-Time and Part-Time Teachers

The weekly working hours and the effort-reward subscales are indicated as work-related characteristics (Table 4). As shown, the full-time teachers in this study taught six lessons (45 min each) more per week on average than part-time teachers. The difference in weekly working hours amounted to 8.5 h. It should be emphasised that the weekly working time in both groups showed a high inter-individual variance (Table 4).

Gender and age had a statistically significant influence on working hours, but this is not practically relevant (η^2^ = 0.001). Regardless of this, female full-time teachers worked around 1.5 h more per week than their male colleagues (∅ 45.7 vs. 44.2 h/week). In contrast, male part-time teacher worked four hours more than their female colleagues (∅ 39.9 vs. 35.9 h/week). Regardless of whether they worked part-time or full-time, younger colleagues (20–29 years) had significantly longer working hours (FT-T vs. PT-T: ∅ 47.0 vs. 43.1 h/week) than older colleagues (60–67 years) (FT-T vs. PT-T: ∅ 42.2 vs. 36.1 h/week).

Statistically significant differences between full-time and part-time teachers (*p* < 0.001) can be seen for the ER subscales due to the sample size but these are also of no relevance (η^2^ = 0.001–0.005) (Table 4). The average values for teachers are in the normal range for effort (∅ 10 of 15 points) and reward (∅ 26 of 35 points). For the ER ratio the means are 0.90 and, thus, still outside the range of risk. However, a health risk (ER ratio >1) was evident for no less than one third of the teachers (FT-T: 35%; PT-T: 31%, d = 0.079).

Means and standard deviations for the teacher group with an ER ratio ≤1 is 0.7 ± 0.2 and for the teacher group with an ER ratio >1 is 1.3 ± 0.3.

### 3.3. Personal Characteristics for Full-Time and Part-Time Teachers

There is no relevant difference between full-time and part-time teachers on average in terms of their ability to recover and overcommitment (Table 5; d = 0.08–0.05). The means of 17 points for both recovery ability and overcommitment can be classified as normal in both groups [23,47], but they are close to the noticeable range (>18 of 24 points). About one third of the teachers (FT-T: 38%; PT-T: 34%) was unable or very unable to recover after work. Additionally, a critical OC value (>18 points) could be observed in 39% of all teachers studied, which can be associated with an increased health risk. The inability to recover and the tendency to overcommit were influenced by a small gender effect (*p* < 0.001; η^2^ = 0.014–0.025). Accordingly, more women (IR: 39%, OC: 43%) than men (IR: 29%, OC: 32%) suffered from an inability to recover and overcommitment. Age did not have an effect on these results (*p* = 0.001–0.500; η^2^ < 0.010).

From the perspective of personal characteristics, there is a clear health risk for about a quarter of teachers (27%) due to an inability to recover and overcommitment. Only half of the teachers (52%) showed normal recovery and commitment values.

### 3.4. Relationship between Work-Related and Personal Characteristics, and Burnout Risk in Teachers

The weekly working hours do show a very small correlation with the burnout risk (r = 0.10). The effort-reward subscales correlate slightly with the burnout risk (r = −0.35–0.42). However, there is a trend that high effort, low reward and an increasing effort-reward imbalance correlate with an increase in burnout risk (Table 6).

For the personal characteristics, there is a somewhat stronger correlation with the risk of burnout (r = 0.44–0.49). An inability to recover and a tendency to overcommit increase the risk of burnout. The covariates (gender, age) have no influence on these correlations (r = 0.01–0.02).

The association between emotional exhaustion, the main component of burnout on the one hand, and work-related and personal characteristics on the other hand seem somewhat clearer (r = 0.09–0.43; IR: r = 0.51–0.55; OC: r = 0.44–0.49) (Table 6).

### 3.5. Predictors of Burnout Risk in Teachers

The simple linear regression shows that—with the exception of weekly working hours—all other work-related and personal characteristics had a statistically significant influence on the burnout risk (Table 7). This means that the variable weekly working hours did not explain the burnout risk among teachers, neither did the control variables gender, age and the extent of employment (full-time vs. part-time); they were, therefore, excluded from further analyses. The ER ratio alone explained 17% of the variance in burnout risk. However, the inability to recover (24%) and a tendency to overcommit (19%) did offer a better explanation of the burnout risk.

The variance explanation of the burnout risk could be improved by the multiple linear regression models. For example, 25% of the variance in the risk of burnout could be explained by the inability to recover and the tendency to overcommit, and 29% by adding the ER ratio. Thus, the ratio of effort and reward, the inability to recover and the tendency to overcommit turned out to be a predictor of the burnout risk. Collinearity had to be observed between the inability to recover and the tendency to overcommit (r = 0.77). Nevertheless, these three predictors represented an optimal combination of characteristics to predict the burnout risk in teachers. This prediction could be estimated using the following regression equation: Burnout risk = −0.58 + 0.24 × ER ratio + 0.21 × IR score + 0.14 × OC score.

## 4. Discussion

Due to complex work requirements, teachers are more exposed to work stress and stress-related health effects, including burnout, than other occupational groups [7,61]. This can have a direct impact on the quality of teaching and the learning success of students [62,63,64]. It is, therefore, important in many respects to keep teachers healthy.

The aim of this study was to examine the relationships between work-related and personal characteristics and the burnout risk of upper-level secondary schoolteachers. The results are at the same time a current status of the burnout risk of German full-time and part-time teachers at these schools since representative data on such prevalence are missing.

One principal finding of the study is that personal characteristics are more important in explaining the burnout risk of teachers than work-related characteristics. Overall, 29% of the burnout risk can be explained by the combination of an inability to recover, overcommitment and the effort-reward ratio. The inability to recover turns out to be the strongest predictor (24%) in explaining burnout risk. In contrast, neither the total weekly working time nor the control variables of gender and age contribute to the explanation of the burnout risk for teachers.

With the differentiated recording of working hours over four weeks, which also covered all extracurricular tasks, it can be shown that part-time teachers work almost nine hours less on average per week than their full-time colleagues. In addition to the lower workload, part-time teachers would thus also have potentially more leeway to recover from the demands of work. Nevertheless, no relevant differences between the two employment groups could be demonstrated in the perceived work-related stress (ER ratio < 1) or in the individual burnout dimensions. The lower workload of part-time teachers, therefore, does not lead to a gain in health. This finding is consistent with the results of previous studies, in which, however, full-time and part-time teachers were only differentiated on the basis of their compulsory teaching hours and the real number of weekly working hours was not known [25].

It can be assumed that part-time teachers can or do not use the comparatively high proportion of free time for recovery. This assumption is supported by the fact that 80% of the part-time workers are female and have children to look after in their own households much more often than their full-time colleagues (73% vs. 35%, *p* < 0.001). At the same time, the Sixth European Working Conditions Survey again provides evidence that care responsibilities and unpaid housework are unevenly distributed between women and men, and that women work more hours overall if both paid and unpaid working hours are taken into account [65]. The explanation is not new: as early as 1991, Byrne claimed that explicitly gender was a background variable for the development of burnout among teachers, in addition to the type of school and age [66].

In later studies, it has been repeatedly claimed that possible effects from work-life conflicts must be considered when interpreting comparatively high values for emotional exhaustion in women [67]. However, time spent with the family can have the opposite effect and reduce the risk of burnout, as shown by Ptáček et al. [68] in a representative survey of Czech teachers. In the data presented here, however, no direct relation between gender and burnout risk or the individual burnout subscales could be determined.

Independent of the time to recover, the present study also examined the individual ability of teachers to recover. More than one third of the participants do not succeed in sufficiently recovering from work-related stress in their free time. As indicated above, the inability to recover has the highest explanatory value for burnout risk and is therefore regarded as an important early indicator of the development of burnout.

Sonnentag and Fritz [41] argued that recovery is only possible when there is sufficient psychological detachment from work during non-work time. This includes the omission of work-related activities as well as the mental detachment from work during non-work time [69]. However, employees who are exposed to significant stressors in the workplace (such as teachers) have less ability to psychologically detach themselves from work, al-though they have a special need for relaxation or recovery [70]. The stressor-detachment model attributes this to the high level of sustained activation triggered by the job stressors [71]. The tendency to not solve this problem can develop into a habit if the employee is often busy with work in the evenings and on days off. The consequence is a chronically elevated level of stress [41].

It is precisely at this point that there is a need for action since teachers’ work conditions are characterised by a number of peculiarities that make effective recovery difficult: long working days and a fragmentation of the work and recovery phases due to different work locations (school/home), regular work in the evenings and at the weekends, as well as a delimitation of work. In the study by Felsing et al. [72], 75% of teachers regularly worked seven days a week. Combinations of longer recovery phases, with the exception of holidays were seldom. The special working time structure in the teaching profession may therefore restrict effective recovery and is even seen as a health risk factor [73].

Regardless of an individual’s ability to recover, the results of this study impressively show that the mental health of a not inconsiderable number of the teachers examined here is at risk. Almost half of the teachers (47%) report burnout symptoms and 3% of them have evidence of burnout. One in four teachers is emotionally exhausted, one in five develops a cynical attitude towards the students they are entrusted with, and one in six feels restricted in their performance. In addition, one third of teachers reports work-related stress in the form of an effort-reward imbalance. In the current study, this ratio, at 0.90, is significantly less favourable than in previous studies (0.64–0.79) on German teachers [25,37,38,74]. In the study by Unterbrink et al. [25], every fifth teacher exceeded the critical ERI cut-off of 1 and was therefore classified as endangered in the long term. In the study presented here, this affected every third teacher, regardless of whether they work part-time or full-time.

The imbalance between high effort and low reward increases the risk of burnout development (r = 0.42; explanation of variance 17%). The tendency to overcommit was also identified as a predictor of burnout risk (variance explanation: 19%). As the overcommitment level increases, so does the proportion of teachers with burnout symptoms (r = 0.49). Among those who already have signs of burnout, almost 80% showed an excessive tendency to overcommit. The results are in line with previous studies predicting burnout risk from overcommitment [75,76].

Overcommitment is supposed to occur particularly in people who are characterised by excessive engagement and the desire for control in demanding situations [27]. In the present study, 39% of the 12,014 teachers showed this coping pattern. These highly overcommitted teachers have an increased health risk in the medium and long term. While the direct effect of overcommitment on health has been robustly proven [27], it remains unclear whether overcommitment also has a moderating effect on the effort-reward ratio.

The average OC value determined for the entire sample at 18 points is close to the cut-off value for health risk (19 points) and seems high compared with the results of a representative cohort study in 40- to 54-year-old workers (∅ 15 points) from a wide variety of occupational groups [77].

In summary, the identified variance explanation of the predictors suggests that burnout is influenced by further characteristics that were not examined here. In particular, physical health and characteristics such as social support and family pressures that (can) affect the mental health of employees were not investigated. In the context of the scope of employment, the results support the thesis that part-time teachers tend to overcommit and thus self-exploit themselves.

The special feature of the present study is that, for the first time, data on the working hours, working conditions and mental health of full-time and part-time upper-level se-condary schoolteachers for Germany as a whole could be presented, taking into account key influencing factors. Compared to other teacher studies, the random sample in the study was characterised by a homogeneous collective. A mixture of teachers with functionaries (staff councils) and school principals was consistently avoided.

The LaiW study also represents a suitable comparison sample for future studies on psycho-social workloads and the psychological situation of healthy teachers and can be used for comparison for return-to-work studies.

The limitations of this study must be taken into account when interpreting the results. The cross-sectional design of this study does not allow a causal interpretation of the reported relationships. Additionally, the results must be interpreted carefully since the study only covered around 11% of all German upper-level secondary schoolteachers. However, the sample was sufficiently large overall and, with some restrictions, was fairly representative with respect to gender and age.

The proportion of male teachers in the full-time group was 8% lower than the proportion of all male, full-time, upper-level secondary schoolteachers in Germany. Additionally, part-time employees participated more frequently than their corresponding share of all upper-level secondary schoolteachers (49% instead of 38%).

With regard to the interpretation of the burnout subscales as “high” or “low” values, it must be noted that they were based on standard values from a sample of US teachers [52], whose response behaviour cannot serve as an internationally valid measure. Such a comparison only provides relative information. As long as binding standard samples for particular populations and especially cut-off values are missing, the findings for general categories such as “burned out” or “at risk of burnout” must be interpreted with caution.

## 5. Conclusions and Outlook

A significant proportion of the upper-level secondary schoolteachers examined here show symptoms of burnout or risk factors for the development of burnout, regardless of the extent of employment, age and gender. The results show that an imbalance between effort and reward, overcommitment and an inability to recover play major roles.

Teachers need modern occupational health and safety. To meet the challenges of work hazardous to health, a combination of organisational measures and person-based prevention approaches is required.

Workloads in schools can be reduced if teachers teach smaller classes with a greater homogeneity of student performance. In addition, teachers need to be better trained in dealing with students with behavioural problems and regularly supported by professional specialists (e.g., school psychologists and social workers).

Working conditions—also for teachers—must be designed in such a way that sufficient recovery is guaranteed. This includes a balance between workload and resources as well as recreational breaks at school and the promotion of sufficiently long recovery phases in the evenings and at weekends. For example, it is essential to create a sufficient number of retreat rooms and quiet workplaces in schools so that during free periods or directly after classes teachers can complete work they would otherwise take home. This could make it easier for some teachers to distance themselves from work and, thus, improve the conditions for recovery.

It is particularly important to recognise teachers at risk in good time, for example in the context of occupational health care. In this context, the acquisition of the inability to recover can serve as an early indicator.

It is particularly important to identify teachers who are at risk at an early stage before they develop burnout syndrome. As with other occupational groups, teachers need preventive occupational health care services at regular intervals. Within this context, the inability to recover could be recorded as an early indicator of burnout risk. In addition, with the relatively simply constructed subscales of the MBI-GS, persons can be identified who feel overburdened by the demands of the teaching profession. Teachers affected by the inability to recover should be taught clear demarcation strategies and techniques for regeneration as part of individual prevention programmes.

## Figures and Tables

**Table 1:** Characteristics of the sample.

Baseline Characteristic	Full-Time Teacher (*n* = 6109)		Part-Time Teacher (*n* = 5905)	
	%	*n*	%	*n*
**Gender**				
male	43.9	2680	19.0	1124
female	56.1	3429	81.0	4781
**Age group [years]**				
25–29	10.4	636	3.6	211
30–39	40.0	2442	28.5	1684
40–49	25.1	1535	41.5	2451
50–59	19.3	1177	22.3	1317
60–67	5.2	319	4.1	242
**Subject combination**				
languages	16.2	990	23.1	1365
social sciences	3.6	220	2.2	127
natural sciences	21.4	1310	19.0	1119
languages and social sciences	24.5	1497	24.3	1436
languages and natural sciences	6.1	373	7.3	431
social sciences and natural sciences	7.7	471	6.0	357
art, music, sports	2.1	126	1.5	90
subject combinations with art, music, sports	18.4	1122	16.6	980
**Family obligation**				
permanent partnership	81.2	4958	88.6	5230
children in the household	34.8	2127	73.2	3731
care of relatives	5.2	318	6.5	383

Comments: %: frequencies; *n*: number.

**Table 2:** Main effects of burnout subscales and covariates of full-time and part-time teachers.

Burnout Subscale	Full-Time Teacher (*n* = 6109)	Part-Time Teacher (*n* = 5905)	F-Value	*p*-Value	Partial Eta^2^
	M ± SD	M ± SD			
**Emotional exhaustion (EE)**	2.4 ± 1.3	2.3 ± 1.2	27.68	<0.001	0.002
gender			55.02	<0.001	0.005
age group			0.83	0.364	<0.001
**Cynicism** **(CY)**	1.3 ± 1.2	1.2 ± 1.1	6.60	0.010	0.001
gender			43.85	<0.001	0.004
age group			39.67	<0.001	0.003
**Personal accomplishment (PA)**	4.9 ± 0.8	4.8 ± 0.8	45.85	<0.001	0.004
gender			3.16	0.076	<0.001
age group			54.32	<0.001	0.005

Comments: M ± SD: mean ± standard deviation; univariate analyses of variance, F-value: test size; *p*-value: significance (two-sided); η^2^: partial eta square (effect size): <0.010 = no effect [59]; corrected R-squared: EE = 0.005; CY = 0.008; PA = 0.008.

**Table 3:** Main effects of burnout risk and covariates and burnout classification of full-time and part-time teachers.

Characteristic	Dimension	Full-Time Teacher (*n* = 6109)	Part-Time Teacher (*n* = 5905)	Test Value	*p*-Value	Effect Size
**Burnout risk (BU-R)**	M ± SD	1.7 ± 0.9	1.6 ± 0.9	F = 4.58	0.032	η^2^ ≤ 0.001
gender				F = 4.88	0.027	η^2^ ≤ 0.001
age group				F = 0.82	0.364	η^2^ ≤ 0.001
**Burnout classification** [48]						
no burnout symptoms	% (n)	49.3 (3011)	49.7 (2933)	χ^2^ = 5.81	0.055	d = 0.044
some burnout symptoms	% (n)	46.9 (2865)	47.3 (2794)			
burnout	% (n)	3.8 (233)	3.0 (178)			

Comments: M ± SD: mean ± standard deviation; univariate analyses of variance, F: test size; η^2^: partial eta square (effect size): <0.010 = no effect [59]; corrected R-squared: BU-R = 0.001. **%**: frequency in %, chi-square test (Pearson), χ^2^: test size; *p*-value: significance (two-sided); d: effect size: d < 0.02 = no effect [59].

**Table 4:** Main effects of the work-related characteristics of full-time and part-time teachers.

Work-Related Characteristic	Dimension	Full-Time Teacher (*n* = 6109)	Part-Time Teacher (*n* = 5905)	F-Value	*p*-Value	Effect Size
Teaching [h/week]	M ± SD	22.6 ± 3.4	16.8 ± 4.3	10717.21	<0.001	η^2^ = 0.472
Working time [h/week]	M ± SD	45.2 ± 8.7	36.7 ± 9.7	2156.11	<0.001	η^2^ = 0.152
Effort [5–15 pts]	M ± SD	9.6 ± 2.6	9.5 ± 2.6	15.66	<0.001	η^2^ = 0.001
Reward [7–35 pts]	M ± SD	26.0 ± 5.4	26.7 ± 5.1	53.53	<0.001	η^2^ = 0.004
ER ratio [0.2–5.0]	M ± SD	0.93 ± 0.43	0.88 ± 0.37	54.56	<0.001	η^2^ = 0.005
ER ratio ≤1	% (n)	65.3 (3990)	69.0 (4075)	18.59^x^	<0.001^x^	d = 0.079
ER ratio >1	% (n)	34.7 (2119)	31.0 (1830)			

Comments: ER ratio: effort-reward ratio; pts: points; M ± SD: mean ± standard deviation; **%** (n): frequency in %, n: number of teachers; χ^2^-test (Pearson), χ^2^: test size; univariate analyses of variance, method; design: constant term + gender + age group; F-value: test size; *p*-value: significance (two-sided); η^2^: partial eta square (effect size): < 0.010 = no effect [59]; d: effect size: <0.1 = no effect, 0.2–0.4 = small effect, 0.5–0.7 = medium effect; corrected R-squared: effort = 0.015, reward = 0.005, ER ratio = 0.010. There are neither gender nor age effects on the effort-reward subscales (η^2^ < 0.01).

**Table 5:** Main effects of personal characteristics of full-time and part-time teachers.

Personal Characteristic [Range]	Dimension	Full-Time Teacher (*n* = 6109)	Part-Time Teacher (*n* = 5905)	F-Value	*p*-Value	Effect Size
**Inability to recover (IR)** **[6–24 pts]**	M ± SD	16.9 ± 3.5	17.2 ± 3.4	13.24	<0.001	η^2^ = 0.007
normal (6–18 pts)	% (n)	62.4 (3812)	65.8 (3887)	20.91 ^x^	<0.001 ^x^	d = 0.084
noticeable (19–20 pts)	% (n)	18.6 (1136)	18.1 (1071)			
very noticeable (≥21 pts)	% (n)	19.0 (1161)	16.0 (947)			
**Overcommitment (OC)** **[6–24 pts]**	M ± SD	17.4 ± 3.4	17.5 ± 3.4	41.01	<0.001	η^2^ = 0.003
normal (6–18 pts)	% (n)	59.6 (3640)	61.8 (3649)	6.15 ^x^	0.013 ^x^	d = 0.045
high (≥19 pts)	% (n)	40.4 (2469)	38.2 (2256)			

Comments: pts: points; M ± SD: mean ± standard deviation; **%** (*n*): frequency in %, n: number of teachers; ^x^: χ^2^-test (Pearson), χ^2^: test size; univariate analyses of variance, method; design: constant term + gender + age group; F-value: test size; *p*-value: significance (two-sided); η^2^: partial eta square (effect size): <0.010 = no effect [59]; d: effect size: <0.10 = no effect; corrected R-squared: IR = 0.023, OC = 0.026. There are no age effects for IR and OC (η^2^ = <0.01) but small gender effects for IR (η^2^ = 0.014) and OC (η^2^ = 0.025).

**Table 6:** Bivariate and partial correlations between work-related and personal characteristics and the burnout risk of teachers (*n* = 12,014).

Characteristic	Bivariate Correlation	Partial Correlation
	Burnout Risk	Controlled Variables (Gender + Age Group)
**Work-related characteristic**		
teaching [h/week]	0.06	0.07
working time [h/week]	0.10	0.10
effort [pts]	0.36.	0.36
reward [pts]	−0.35	−0.35
effort-reward ratio	0.42	0.42
**Personal characteristic**		
inability to recover [pts]	0.49	0.49
overcommitment [pts]	0.44	0.44

Comments: Bivariate and partial correlations: correlation coefficient r (Pearson-Bravais). 0.00 < r <0.10 = no correlation, 0.01 < r ≤0.20: very small correlation, 0.20 < r ≤0.50: small correlation [60].

**Table 7:** Regression models of work-related and personal characteristics with burnout risk in teachers (n = 12,014).

Model		Standardized Coefficients B	t-Value	*p*-Value	95% CI for B		Corrected R^2^
					Lower Limit	Upper Limit	
	**Work-related characteristics**						
1	constant		39.46	<0.001	1.23	1.36	
	working time	0.10	10.82	<0.001	0.01	0.01	0.010
2	constant		46.47	<0.001	0.79	0.86	
	ER ratio	0.42	50.13	<0.001	0.87	0.94	0.173
3	constant		24.51	<0.001	0.72	0.84	
	working time	0.02	1.75	0.080	0.00	0.00	0.173
	ER ratio	0.41	48.75	<0.001	0.86	0.93	
	**Personal characteristics**						
4	constant		−11.70	<0.001	−0.47	−0.33	
	IR score	0.49	61.07	<0.001	0.12	0.12	0.237
5	constant		−8.03	<0.001	−0.37	−0.22	
	OC score	0.44	53.60	<0.001	0.11	0.12	0.193
6	constant		−16.05	<0.001	−0.67	−0.52	
	IR score	0.36	29.55	<0.001	0.08	0.09	0.248
	OC score	0.16	13.12	<0.001	0.04	0.05	
	**Total model**						
7	constant		−16.08	<0.001	−0.65	−0.51	
	ER ratio	0.24	27.37	<0.001	0.48	0.55	0.292
	IR score	0.27	21.60	<0.001	0.06	0.07	
	OC score	0.14	11.92	<0.001	0.03	0.04	

Comments: ER ratio: effort-reward ratio; IP score: total value of inability to recover; OC score: total value of overcommitment; dependent variable: burnout risk; simple and multiple linear regression (method: inclusion), CI: confidence interval; t-value: test size; *p*-value: significance (two-sided).

## Data Availability

The data are not publicly available due to agreed data protection commitments to the participants.
